# Characteristics of HIV Infection in Paediatric Admissions to a Rural Hospital in Zaire

**Published:** 1990-09

**Authors:** Rebecca Park

**Affiliations:** Fifth Year Medical Student, Bristol University


### Characteristics of HIV Infection in Paediatric Admissions to a Rural Reference Hospital in Bas-Zaire, July 1986–November 1989

**Rebecca Park**
Fifth Year Medical Student, Bristol University

### INTRODUCTION

HIV infection is a growing problem worldwide. The epidemic is more advanced in Africa than in the western world. Over 50% of the estimated 6,000,000 people infected are in Africa. Here the epidemic is heterosexual, and children suffering from congenital infection form the fastest-growing proportion of cases. The characteristics and prevalence of HIV infection in children remain largely unexplored, especially in areas of rural Africa. Longitudinal studies are underway, but have not yet yielded many results. Kimpese is a town of 28,000 in lower Zaire, on the main road between Kinshasa, 220 km, and the principal port of Matadi, 140 km. The hospital of IME, established in 1952, is a joint enterprise of protestant churches in Zaire, Europe and North America. It is the official government reference hospital for the rural health area of Kimpese, with a population of 120,000. It also acts as a wider reference centre for lower Zaire. The hospital has 365 inpatient beds, including 50 paediatric beds. In 1986 there were 1,658 paediatric admissions, in 1987 there were 1335, and in 1988, 1212.

### METHODS

During my 10-week elective at IMÉ, Kimpese, I saw HIV infection in children at various ages and stages of infection, in outpatients and as admissions. In an attempt to glean some idea of any characteristics of such infections over the past few years, I surveyed the paediatric admissions register, records of which were available from the latter half of 1986.

### RESULTS

Paediatric records document approximate age, date of admission, and principal complaints of each case. Patients with HIV infection often present with symptoms of other common diseases, thus HIV may remain undetected as the cause of their illness. However, paediatrician Dr Stephen Green has recognised cases of HIV infection since 1985, and has since then utilised the same index of suspicion when ordering the HIV status of a child to be investigated. An MRC-funded longitudinal study, Project Nkembolo, underway at present at IMÉ follows a cohort of 80 children born to HIV-positive mothers and 50 controls born to HIV-negative mothers in an attempt to define characteristics of the disease in children. HIV-antibody testing is thus available and reliable at IMÉ. The results expressed below represent children other than those involved in the above study, which commenced in July 1988.

### DISCUSSION

Figure 1 shows a trend towards increasing numbers of admissions for HIV over the past 3 years. The proportion of deaths seems to have fallen, possibly due to earlier recognition and treatment of AIDS, and/or earlier presentation. Many project children admitted during my stay (Oct-Nov 1989) are not included in these figures, as their HIV status is blinded. They therefore provide a bias to the total admissions especially in the past year, as over half of them may be suffering HIV-related disease.

Figure 2 tabulates the major complaints of the admissions. Many children with HIV-related disease demonstrate multiple pathology, but by far the commonest presentations in this region seems to be TB and malnutrition, often simultaneously. The category "AIDS" represents a non-specific, often terminal, state, a label given mainly to the large proportion of children dying from terminal HIV infection, presenting at a late stage, in 1986–87. A larger proportion of children over the last 2 years have survived, and a larger proportion are given more specific diagnoses. This may be a source of observer error, and again increasing recognition.

Figure 3 tabulates the age distribution of the admissions. It can be seen that the peak age incidence is in the first 2 years of life. Indeed it appears that there may be a high incidence in the first 6 months of life, and a second peak of presentation from 12 to 24 months of age. This pattern may bear a relation to decline in placentally transmitted maternal antibodies, and the role of breast-feeding. Interestingly, there seems to be a third smaller peak around 4 years of age, although with such a small sample size one cannot come to any firm conclusions. Also the data available is not specific as to exact age, i.e. some childrens' ages were expressed in months and some in years, the latter being a less specific measure. The results shown are necessarily crude, but demonstrate patterns which it will be interesting to follow with more extensive and accurate studies.

### REFERENCES

GREEN, S. G., Protocol, MRC funded project: "The natural history of HIV infection of children in rural Zaire." (1988)

### ACKNOWLEDGEMENTS

Dr. Stephen Green, the paediatric staff of IMÉ, and Professor David Park.

